# Arrhythmia prevention protocol during papaverine administration for invasive coronary functional assessment

**DOI:** 10.3389/fcvm.2025.1621053

**Published:** 2025-09-18

**Authors:** Dobrin Vassilev, Niya Mileva, Gavril Stoev, Ilter Pazardzhykly, Panayot Panayotov, Gianluca Rigatelli, Valery Gelev

**Affiliations:** ^1^ SHATC Medica Cor, Ruse, Bulgaria; ^2^ Ruse University “Angel Kanchev”, Ruse, Bulgaria; ^3^ Medical University of Pleven, Pleven, Bulgaria; ^4^ Ospedali Riuniti Padova Sud, Padova, Italy; ^5^ Cardiology Clinic, Acibadem City Clinic Tokuda Hospital, Sofia, Bulgaria

**Keywords:** arrhythmia, functional assessment, fractional flow reserve, chronic coronary syndrome, hyperemia

## Abstract

**Background:**

Accurate assessment of coronary physiology through invasive functional testing is critical for the effective management of chronic coronary syndromes. The induction of maximal and sustained coronary hyperemia is essential for obtaining reliable measurements of fractional flow reserve (FFR), coronary flow reserve (CFR), and the index of microcirculatory resistance (IMR). Intracoronary papaverine is a potent vasodilator for inducing hyperemia but has been associated with ventricular arrhythmias, limiting its clinical use.

**Methods:**

This single-center prospective study investigates the feasibility and safety of a novel hyperemic protocol involving intravenous (i.v.) lidocaine administration followed by intracoronary papaverine in patients undergoing invasive coronary functional assessment. All patients underwent FFR, CFR, and IMR measurements after receiving an initial dose of 100 mg of i.v. lidocaine followed by papaverine (20 mg for the left coronary artery and 5–10 mg for the right coronary artery).

**Results:**

A total of 389 patients were enrolled. Functionally significant stenosis (FFR ≤ 0.80) was identified in 36% of patients and microvascular dysfunction in 48%. Ventricular arrhythmias occurred in 1.5% of patients, including four episodes of ventricular fibrillation and two of ventricular tachycardia; all resolved with prompt defibrillation and without hemodynamic compromise.

**Conclusion:**

These findings suggest that pre-treatment with lidocaine may enhance the safety of papaverine-induced hyperemia during invasive coronary testing.

## Introduction

Comprehensive invasive functional assessment in the catheterization laboratory is an important part of the guideline-directed management of patients with chronic coronary syndromes (1). The induction of both maximal and steady-state coronary hyperemia is a mandatory step during the measurement of hyperemic indexes such as fractional flow reserve (FFR), coronary flow reserve (CFR), and index of microcirculatory reserve (IMR). Ideally, an adequate vasodilator should have the following characteristics: (i) the coronary vasodilation should be maximal; (ii) the agent should have a rapid-onset and steady-state effect and a short duration of action, to regulate the duration of maximal hyperemia; and (iii) the agent should have no significant side effects. Failure to meet any of these conditions could lead to incorrect clinical decisions. Previous studies have shown that intracoronary papaverine is the most potent pharmacological vasodilator, producing the greatest increase in coronary blood flow, enabling stable and prolonged hyperemic induction (2–4). However, this drug has been reported to increase the risk of ventricular arrhythmias. Previous studies reported rates ranging from 1.7% to 4.5%, while larger series reported lower rates, between 0.7% and 2.7%, depending on population and methodology. Torsades de pointes (TdP) was the most frequently occurring rhythm disturbance, more common in women than in men (4.4% vs. 0.3%), but severe sinus bradycardia has also been described (5).

## Methods

We conducted a single-center prospective study to evaluate the feasibility and safety of a hyperemic protocol using initial intravenous (i.v.) administration of lidocaine followed by intracoronary papaverine for hyperemic induction in patients undergoing invasive coronary functional assessment. Consecutive patients with chronic coronary syndrome referred for invasive coronary angiography were included. All patients underwent a comprehensive functional assessment with measurements of FFR, CFR, and IMR. The following cutoff values were accepted for abnormal results, i.e., FFR ≤ 0.80, CFR < 2.5, and IMR ≥ 25, according to the latest recommendations (1). All patients received an i.v. bolus of 100 mg lidocaine, administered immediately (<2 min) before intracoronary papaverine administration (20 mg in the left coronary artery and 5–10 mg in the right coronary artery). The rate of arrhythmic events was recorded. All patients were managed following the Declaration of Helsinki and provided informed consent for anonymous publication of scientific data. The study protocol was approved by the local ethics committee (6/15.01.2024).

## Results

A total of 389 patients with chronic coronary syndrome were included in the study. The mean age was 67 ± 9 years, and 69% of the patients were male (Table 1). Dyslipidemia was present in 96% (*n* = 268), 64% (*n* = 249) were smokers, and 29% (*n* = 113) had diabetes mellitus. A history of myocardial infarction was reported in 18% (*n* = 70) and prior percutaneous coronary intervention in 31% (*n* = 70). Peripheral artery disease was present in 6% (*n* = 23), prior stroke in 12% (*n* = 47), and chronic obstructive pulmonary disease (COPD) in 6% (*n* = 23). The mean left ventricular ejection fraction was 55 ± 9. Functionally significant stenosis with FFR ≤ 0.80 was found in 36% of patients (*n* = 140); all of these patients with obstructive coronary artery disease underwent PCI. In 48% of the patients (*n* = 187), microvascular dysfunction was detected with an abnormal CFR and/or IMR. No abnormality was found in the remaining 16% of patients. A total of six (1.5%) ventricular arrhythmic events were recorded after papaverine administration (Table 2). Two patients had an episode of ventricular tachycardia, and four patients had an episode of ventricular fibrillation (VF); these episodes required 200 J biphasic defibrillation. In addition, one episode of atrial fibrillation was recorded and self-terminated. The target lesion in two of the patients with VF was the right coronary artery (RCA), and for the rest, the examined vessel was left anterior descending artery (LAD). All patients recovered sinus rhythm and were hemodynamically stable by the end of the procedure. There were no significant differences in the clinical characteristics of patients with and without arrhythmic events, except that those with arrhythmia had a higher rate of peripheral vascular disease (16% vs. 6%, *p* = 0.032) and lower rates of stroke (0% vs 12%) and COPD (0% vs. 6%, *p* < 0.001). QTc prolongation (>480 ms) was observed in three of the cases with arrhythmia. No lidocaine-related adverse events were observed.

**Table 1:** Patient's demographic and clinical characteristics.

Demographic characteristics	All	No arrhythmia	Arrhythmic event	*p*-value
	*N* = 389	*N* = 383	*N* = 6	
Age, years (mean ± SD)	67 ± 9	67 ± 9	66 ± 9	0.512
Male sex, *n* (%)	268 (69)	262 (69)	4 (70)	0.119
Medical history				
Hypertension, *n* (%)	386 (99)	381 (98)	5 (99)	0.704
Diabetes, *n* (%)	113 (29)	111 (28)	2 (33)	0.098
Dyslipidemia, *n* (%)	373 (96)	368 (96)	5 (83)	0.073
Prior myocardial infarction (MI), *n* (%)	70 (18)	69 (18)	1 (17)	0.236
**Prior stroke, *n* (%)**	**47** **(12)**	**47** **(12)**	**0** **(0)**	**0** **.** **001**
**Peripheral arterial disease, *n* (%)**	**23** **(6)**	**22** **(5.7)**	**1** **(16)**	**0** **.** **031**
Smokers, *n* (%)	249 (64)	246 (65)	3 (50)	0.102
Chronic kidney disease, *n* (%)	70 (18)	69 (18)	1 (18)	0.811
**COPD, *n* (%)**	**23** **(6)**	**23** **(6)**	**0** **(0)**	**0** **.** **001**

Parameters with significant statistical difference between groups are marked in bold.

**Table 2:** Patient's procedural characteristics and arrhythmic events.

Parameter	Value
Procedural characteristics	
Procedural time (mean ± SD)	59 ± 12
Contrast (mean ± SD)	165 ± 19
Mean FFR (mean ± SD)	0.81 ± 0.03
Mean CFR (mean ± SD)	2.4 ± 0.5
Mean IMR (mean ± SD)	21 ± 2
Patients with FFR ≤ 0.80, *n* (%)	140 (36)
Patients with CFR < 2.5, *n* (%)	131 (34)
Patients with IMR > 25, *n* (%)	102 (26)
Coronary microvascular dysfunction, *n* (%)	187 (48)
Arrhythmic events	
Ventricular tachycardia, *n* (%)	2 (0.5)
Ventricular fibrillation, *n* (%)	4 (1.03)
Atrial fibrillation, *n* (%)	1 (0.3)

## Discussion

Previous studies and registries have consistently shown that although intracoronary papaverine is one of the most potent agents for inducing maximal and sustained coronary hyperemia, its use has been limited by concerns regarding its potential for proarrhythmia. Early observational studies highlighted the superior vasodilatory effect of papaverine compared with adenosine and other agents, particularly in achieving stable hyperemic conditions essential for IMR and CFR assessment (6, 7). However, these benefits were tempered by a reported incidence of ventricular arrhythmias ranging from 1.9% to 4.5%, with TdP and VF being the most serious complications, especially in women and in patients with prolonged QT intervals (8). More recent analyses, including data from large registries and multicenter cohorts, have confirmed this risk profile, prompting caution in certain patient populations (9). An additional procedural consideration relates to the potential proarrhythmic effect of inadvertent contrast–papaverine interaction within the infusion system. Previous reports have suggested that residual contrast medium within guiding catheters or extension lines may potentiate the arrhythmogenic risk of papaverine (10). Although this phenomenon was not directly evaluated in our study, all procedures incorporated a standardized protocol involving thorough saline flushing of the catheter system prior to papaverine administration to minimize this risk. Our findings underscore the importance of meticulous catheter preparation and drug delivery technique during invasive coronary functional testing. Our findings support the hemodynamic effectiveness of papaverine while introducing a potential mitigation strategy through pre-treatment with intravenous lidocaine. The arrhythmia rate observed in our study (1.5%) is lower than the one previously reported, suggesting that lidocaine may confer a protective electrophysiological effect, possibly by stabilizing myocardial excitability and reducing dispersion of repolarization. While our findings suggest the potential protective role of lidocaine, the lack of a comparator arm precludes firm conclusions. Therefore, our findings should be interpreted as hypothesis-generating. Further studies are warranted to validate this approach across broader populations and to optimize safety protocols during invasive coronary functional testing.

## Conclusion

The use of a potent and safe hyperemic agent during invasive coronary functional testing represents an unmet clinical need. A protocol of initial lidocaine administration prior to intracoronary papaverine injection may reduce arrhythmic events during invasive coronary functional testing.

## Data Availability

The raw data supporting the conclusions of this article will be made available by the authors, without undue reservation.

## References

[1] Vrints C Andreotti F Koskinas KC Rossello X Adamo M Ainslie J 2024 ESC Guidelines for the management of chronic coronary syndromes. Eur Heart J. (2024) 45(36):3415–537. 10.1093/eurheartj/ehae177 39210710

[2] Wilson RF White CW. Intracoronary papaverine: an ideal coronary vasodilator for studies of the coronary circulation in conscious humans. Circulation. (1986) 73(3):444–51. 10.1161/01.CIR.73.3.444 3948354

[3] Kodeboina M Nagumo S Munhoz D Sonck J Mileva N Gallinoro E Simplified assessment of the index of microvascular resistance. J Interv Cardiol. (2021) 2021(1):9971874. 10.1155/2021/9971874 34149324 PMC8189791

[4] De Bruyne B Pijls NH Barbato E Bartunek J Bech JW Wijns W Intracoronary and intravenous adenosine 5′-triphosphate, adenosine, papaverine, and contrast medium to assess fractional flow reserve in humans. Circulation. (2003) 107(14):1877–83. 10.1161/01.CIR.0000061950.24940.88 12668522

[5] Talman CL Winniford MD Rossen JD Simonetti I Kienzle MG Marcus ML. Polymorphous ventricular tachycardia: a side effect of intracoronary papaverine. J Am Coll Cardiol. (1990) 15(2):275–8. 10.1016/S0735-1097(10)80048-8 2299067

[6] Wilson RF Wyche K Christensen BV Zimmer S Laxson DD. Effects of adenosine on human coronary arterial circulation. Circulation. (1990) 82(5):1595–606. 10.1161/01.CIR.82.5.1595 2225364

[7] Meuwissen M Siebes M Chamuleau SA van Eck-Smit BL Koch KT de Winter RJ Hyperemic stenosis resistance index for evaluation of functional coronary lesion severity. Circulation. (2002) 106(4):441–6. 10.1161/01.CIR.0000023041.26199.29 12135943

[8] Nakayama M Tanaka N Sakoda K Hokama Y Hoshino K Kimura Y Papaverine-induced polymorphic ventricular tachycardia during coronary flow reserve study of patients with moderate coronary artery disease—analysis of ECG data. Circ J. (2015) 79(3):530–6. 10.1253/circj.CJ-14-1118 25746536

[9] Okabe Y Otowa K Mitamura Y Murai H Usui S Kaneko S Evaluation of the risk factors for ventricular arrhythmias secondary to QT prolongation induced by papaverine injection during coronary flow reserve studies using a 4 Fr angio-catheter. Heart Vessels. (2018) 33:1358–64. 10.1007/s00380-018-1175-8 29713819

[10] Kern MJ Deligonul U Serota H Gudipati C Buckingham TJC. Ventricular arrhythmia due to intracoronary papaverine: analysis of QT intervals and coronary vasodilatory reserve. Cathet Cardiovasc Diagn. (1990) 19(4):229–36. 10.1002/ccd.1810190402 2334953

